# Predictors and immunological correlates of sublethal mercury exposure in vampire bats

**DOI:** 10.1098/rsos.170073

**Published:** 2017-04-19

**Authors:** Daniel J. Becker, Matthew M. Chumchal, Alexandra B. Bentz, Steven G. Platt, Gábor Á. Czirják, Thomas R. Rainwater, Sonia Altizer, Daniel G. Streicker

**Affiliations:** 1 Odum School of Ecology, University of Georgia, Athens, GA, USA; 2 Center for the Ecology of Infectious Disease, University of Georgia, Athens, GA, USA; 3 Department of Poultry Science, University of Georgia, Athens, GA, USA; 4 Department of Biology, Texas Christian University, Fort Worth, TX, USA; 5 Wildlife Conservation Society, Myanmar Program, Yangon, Myanmar; 6 Department of Wildlife Diseases, Leibniz Institute for Zoo and Wildlife Research, Berlin, Germany; 7 Tom Yawkey Wildlife Center and Belle W. Baruch Institute of Coastal Ecology and Forest Science, Clemson University, Georgetown, SC, USA; 8 Institute of Biodiversity, Animal Health and Comparative Medicine, University of Glasgow, Glasgow G12 8QQ, UK; 9 MRC-University of Glasgow Centre for Virus Research, Glasgow G61 1QH, UK

**Keywords:** agriculture, Chiroptera, ecoimmunology, ecotoxicology, spatio-temporal, wildlife health

## Abstract

Mercury (Hg) is a pervasive heavy metal that often enters the environment from anthropogenic sources such as gold mining and agriculture. Chronic exposure to Hg can impair immune function, reducing the ability of animals to resist or recover from infections. How Hg influences immunity and susceptibility remains unknown for bats, which appear immunologically distinct from other mammals and are reservoir hosts of many pathogens of importance to human and animal health. We here quantify total Hg (THg) in hair collected from common vampire bats (*Desmodus rotundus*), which feed on blood and are the main reservoir hosts of rabies virus in Latin America. We examine how diet, sampling site and year, and bat demography influence THg and test the consequences of this variation for eight immune measures. In two populations from Belize, THg concentrations in bats were best explained by an interaction between long-term diet inferred from stable isotopes and year. Bats that foraged more consistently on domestic animals exhibited higher THg. However, relationships between diet and THg were evident only in 2015 but not in 2014, which could reflect recent environmental perturbations associated with agriculture. THg concentrations were low relative to values previously observed in other bat species but still correlated with bat immunity. Bats with higher THg had more neutrophils, weaker bacterial killing ability and impaired innate immunity. These patterns suggest that temporal variation in Hg exposure may impair bat innate immunity and increase susceptibility to pathogens such as bacteria. Unexpected associations between low-level Hg exposure and immune function underscore the need to better understand the environmental sources of Hg exposure in bats and the consequences for bat immunity and susceptibility.

## 1. Introduction

Mercury (Hg) is a pervasive heavy metal with neurotoxic effects in humans and wildlife [1,2]. Hg enters the environment through natural and anthropogenic sources and bioaccumulates as it moves up the food chain, posing particular risks to animals at higher trophic levels [3,4]. For example, high Hg concentrations in livers of piscivorous common eiders (*Somateria mollissima*) were associated with lower weight and smaller spleens [5], indicating altered condition and immune function. Additionally, loggerhead sea turtles (*Caretta caretta*) with high concentrations of blood Hg had fewer lymphocytes and lower B-cell proliferation [6]. These impaired immune responses from Hg exposure can increase host susceptibility to pathogens. As one example, exposure to Hg impaired the resistance of laboratory mice to *Plasmodium vivax* infection [7].

Bats (Order: Chiroptera) are reservoir hosts of many pathogens important to human and animal health [8] and in some areas have suffered population declines caused by white-nose syndrome [9]. However, the consequences of Hg on bat immune function and susceptibility to infection are unknown. The effects of Hg on bat immunity could differ from those in other taxa, as aspects of immune function in bats, such as constitutively expressed interferon, appear unique among mammals [10,11]. Features of bat ecology (high mobility, presence in human-modified habitats) and life history (long lifespan, rapid metabolism, high trophic level) could furthermore increase exposure to and bioaccumulation of heavy metals [12], including Hg [13–15]. Past work has shown adverse effects of chronic Hg exposure on bats, including neurochemical change [16] and mitochondrial DNA damage [17]. However, research on such health effects in bats has almost exclusively been on insectivores in temperate areas of North America and Europe [12]. The distribution and impacts of Hg exposure in bats in the Neotropics has not been assessed, although land conversions such as gold mining can cause extreme Hg contamination [18,19].

The common vampire bat (*Desmodus rotundus*) could be particularly prone to high Hg exposure. Vampire bats occur across diverse habitat types in Latin America from Mexico to Argentina [20]. In much of this range, these bats preferentially feed on livestock, which are more accessibile and reliable food resources than wild prey such as tapir and peccary [21,22]. This can lead to population overabundance in agricultural habitats [23]; however, living in agricultural habitats could come at a cost if bats are exposed to Hg during foraging. Despite being terrestrial herbivores, livestock within agricultural habitat could be contaminated with Hg via atmospheric deposition from point sources such as industrial plants [24], release of naturally occurring Hg from soil by slash-and-burn practices [25], agrochemical use [26] and feed [27]. Vampire bats could accordingly be exposed to Hg through drinking large quantities of livestock blood [18,28]. Chronic Hg exposure might impair bat immunity and increase susceptibility to pathogens, including rabies virus, a major threat to livestock and human health in Latin America [29].

We here examined the predictors and immunological correlates of Hg exposure in vampire bats from agricultural areas of Belize. We first tested the hypothesis that more consistent feeding on livestock or within agricultural habitat increases bat exposure to Hg. We measured stable isotopes of carbon (δ^13^C) and nitrogen (δ^15^N) from hair samples to infer foraging choices across long time spans [30]. Long-term exposure to Hg was also quantified from hair, as metals are sequestered in growing tissue [31], thus providing diet and Hg measures on the same timescale. To test the hypothesis that chronic Hg exposure impairs bat immunity, we next quantified eight measures of immunity from blood samples that span the functions (cellular and humoral) and arms (innate and adaptive) of the vertebrate defence system [32].

## 2. Material and methods

### 2.1. Capture and sampling of vampire bats

**Figure 1.**
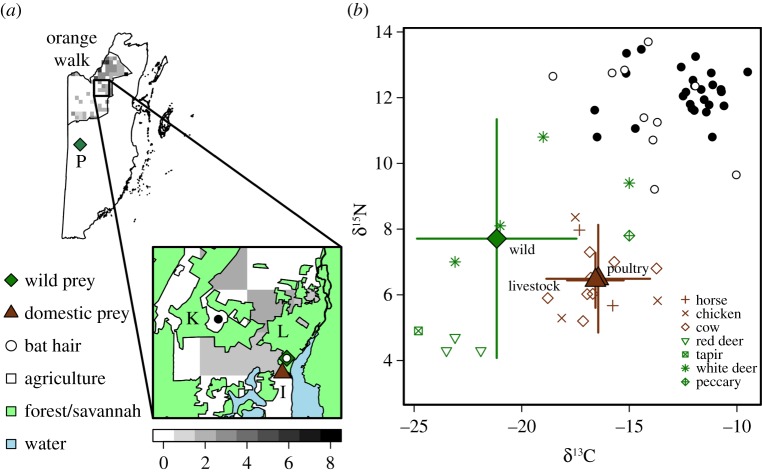
(*a*) Sampling locations of vampire bats and potential prey in Orange Walk District, Belize. Points show the sample location and type (bat and type of prey) in four sites (K, Ka'Kabish; I, Indian Church Village; L, LAR; P, Pacbitun). Grey shading denotes cattle density (per square kilometre) from the Food and Agriculture Organization estimates in each 5 km cell. Polygons display main ecosystem classifications from the Biodiversity and Environmental Resource Data System of Belize, with transparent regions corresponding to agricultural habitat. (*b*) Uncorrected means and standard deviation of δ^13^C and δ^15^N for wild (green diamond) and domestic (livestock and poultry; brown triangle) prey categories. Coloured symbols indicate individual prey isotope values with colours corresponding to prey group (domestic versus wild) and shapes corresponding to species. Circles are isotope values for individual bats (white, LAR; black, Ka'Kabish).

During April–May 2014 and 2015, we sampled vampire bats in two regions of the Orange Walk District of Belize: Lamanai Archaeological Reserve (LAR) and Ka'Kabish (figure 1*a*). Broadleaf deciduous forest borders cattle pastures and agricultural areas cleared by slash and burn [33–35]; cattle densities near these bat colonies range from 0 to 8 km^−2^ [36]. Hg has been detected in wildlife from this region and throughout Belize more broadly, with concentrations exceeding thresholds for adverse health effects in some species [37–39]. Our sites were approximately 8 km apart and consisted of roosts in a hollow tree and cistern (LAR) and looters' tunnels dug into Mayan ruins (Ka'Kabish). Bats were captured with mist nets placed at exits of roosts or along flight paths from 19.00 until 22.00 h. A harp trap was also set from 19.00 until 04.00 h. Bats were placed in individual holding bags and issued uniquely coded incoloy wing bands (3.5 mm, Porzana Inc.). We classified age as sub-adult or adult based on fusion of phalangeal epiphyses [40].

For mercury and stable isotope analyses, we trimmed less than 10 mg of hair from the dorsal posterior region of each bat. To quantify immune function, we collected a maximum of 100 µl of blood by lancing the propatagial vein with a sterile 23-gauge needle, followed by collection using heparinized capillary tubes. Thin blood smears were prepared on glass slides and stained with buffered Wright–Giemsa (Camco Quik Stain II). We obtained plasma by centrifuging blood in serum separator tubes and froze samples at –20°C until –80°C storage at the University of Georgia (UGA). Bats were released at their capture site after processing.

### 2.2. Hair Hg analysis

Bat hair samples were analysed for total Hg (THg). Prior to analysis, hair was rinsed in a 2 : 1 chloroform : methanol solution and dried overnight in a fume hood. We quantified hair THg in all samples with a direct Hg analyser (DMA-80, Milestone, CT, USA), which uses thermal decomposition, gold amalgamation and atomic absorption spectrometry [41]. We analysed National Research Council Canada reference material DORM 4 (certified value = 0.412 ± 0.036 mg kg^−1^) every 10 samples for quality assurance; mean recovery was 88.5 ± 1.65%. Limited amounts of hair available for analysis resulted in many samples falling below the THg detection limit (0.48 ng, approximately 0.096 mg kg^−1^; *n* = 28); these values were subsequently estimated as 50% the detection limit [42]. Hair THg was expressed as mg kg^−1^ and log-transformed prior to analyses.

### 2.3. Stable isotope analysis

Hair samples were dried at 60°C for 72 h and cut into small fragments [22]. Stable isotope signatures were determined using a Thermo Delta V isotope ratio mass spectrometer at the UGA Center for Applied Isotope Studies. Isotope values were expressed in standard *δ* notation, where *δ*^13^C or *δ*^15^N = [(*R*_sample_/*R*_standard_) − 1] × 1000 and *R* is the ratio of ^13^C/^12^C or ^15^N/^14^N). All isotope analyses used two standards for every 12 samples: bovine (δ^13^C = –21.75 ± 0.05 and δ^15^N = 7.49 ± 0.06) and spinach (δ^13^C = –27.53 ± 0.11 and δ^15^N = –0.73 ± 0.26).

Bats feeding on livestock can be differentiated from those feeding on wild prey using δ^13^C [21,22], as most grasses consumed by livestock use the C4 pathway and most forest plants consumed by wildlife use the C3 pathway [43]. In addition, δ^15^N provides inference into trophic position, as consumer δ^15^N is typically enriched by 3−4‰ relative to its diet [44]. We collected samples from potential domestic animal prey in Indian Church Village (figure 1*a*), including hair from two horses (*Equus caballus*) and eight cows (*Bos* spp.) and feathers from three chickens (*Gallus domesticus*). For potential wildlife prey, we used data from archaeological remains of three red deer (*Mazama americana*) and one tapir (*Tapirus bairdii*) collected at LAR [45] and of five white-tailed deer (*Odocoileus virginianus*) and one collared peccary (*Tayassu tajacu*) collected at Pacbitun in the adjacent Cayo District [46]. We applied a correction of –1.5‰ to convert archaeological δ^13^C to modern δ^13^C [47]. We next used Bayesian stable isotope mixing models with the *siar* package in R to quantify the contribution of domestic animal prey to bat isotope signatures [48,49]. Prey were collapsed into domestic animal (livestock and poultry) and wildlife groups, which differed in δ^13^C (*F*_1,21_ = 16.75, *p* < 0.001) but not δ^15^N (*F*_1,21_ = 1.44, *p* = 0.24; figure 1*b*). We used trophic fractionation factors and standard deviations of hair from another phyllostomid, *Leptonycteris yerbabuenae* (2.7 ± 0.2‰ for δ^13^C and 3.3 ± 1.5‰ for δ^15^N; [50]). We ran models with 200 000 iterations, removing 20 000 for burn-in and thinning by a factor of 90 [51]. The 2000 results per bat were then averaged to provide the mean proportion of domestic animals (per cent livestock and poultry) incorporated into an individual's diet.

### 2.4. Leucocyte profiles

We used leucocyte profiles derived from blood smears to measure investment in cellular immunity and chronic stress. From each blood smear, we quantified the total white blood cell (WBC) count as the average number of leucocytes from 10 fields of view at 400× magnification using a light microscope. Differential WBC counts recorded the number of neutrophils, lymphocytes, monocytes, eosinophils and basophils out of 100 leucocytes under 1000× magnification. One observer (D.J.B.) performed all haematological analyses. Absolute numbers of each leucocyte were obtained by multiplying differential and total counts [52], and absolute leucocyte counts were quarter-root transformed for analyses. We calculated the ratio of neutrophils to lymphocytes (i.e. NL ratio) as a measure of chronic stress [53]. Although NL ratios are less sensitive to capture stress than glucocorticoid hormones, they can increase in hours to days after a stressor; however, we found no effect of holding time (*F*_1,13_ = 0.04, *p* = 0.84, *n* = 15).

### 2.5. Bacterial killing assay

We measured innate immune defence by quantifying the *ex vivo* bacterial killing activity of plasma against *Escherichia coli* ATCC 8739 [54], which is killed in plasma predominantly through complement proteins [55]. We used the microplate reader method [56], in which plasma dilutions in sterile phosphate buffered saline (PBS) were optimized to 1 : 8 to kill 50% of *E. coli* (E power Microorganisms no. 0483E7, Microbiologics Inc.). Test samples were run in 22 µl duplicates and challenged with 5 µl of a 10^4^ bacteria ml^−1^ solution in PBS. We prepared tryptic soy broth (TSB; Bacto, BD) 2 days prior to each assay [57] and added 125 µl TSB to each well. Optical density (OD) was measured at 340 nm to determine background OD prior to bacterial growth and again after incubation for 12 h at 37°C. To quantify bacterial killing ability (BKA), we subtracted background OD from OD at 12 h and calculated BKA as one minus mean OD per sample, divided by mean OD of positive controls (5 µl bacteria and 22 µl PBS). BKA of 100% thus represents complete clearance of *E. coli* from the challenge.

We used beta regression models with the *betareg* package to test for effects of capture stress and plasma storage time on BKA, applying a linear transformation to bound proportions to the open unit interval [58,59]. BKA was not affected by holding time (*R*^2^ = 0.05, *p* *=* 0.41, *n* = 14) nor storage time of plasma at –80°C (*R*^2^ = 0.04, *p* = 0.37, *n* = 19). The intra-assay coefficient of variation (CV) was 6.2%, although inter-assay CV was somewhat large (17.4%). We, therefore, included assay plate as a covariate to model the beta regression precision parameter (*ϕ*) in BKA analyses to account for variable dispersion [60].

### 2.6. Immunoglobulin G antibody

We quantified overall immunoglobulin G (IgG) antibody in plasma using a protein G ELISA [61]. We diluted 3 μl of each plasma sample to 1 : 30 000 with 50 mM NaHCO_3_ buffer at pH 9.5 and ran 100 µl of each diluted sample in duplicate using protein G–horseradish peroxidase conjugate (P21041, Life Technologies). According to the Beer–Lambert Law, antibody concentration is proportional to OD (measured at 450 nm). We analysed mean OD of duplicates.

Linear regression showed no relationship between IgG OD and bat holding time (*F*_1,13_ = 0.57, *p* = 0.46). IgG OD was slightly lower in samples frozen for 2 years, but this relationship was not statistically significant (*F*_1,18_ = 3.43, *p* = 0.08). The intra-assay CV was 1.5%, yet inter-assay CV was large (69.2%). We thus included assay plate as a random effect in IgG OD analyses to account for this variation [62].

### 2.7. Statistical analysis

Although we measured hair THg in 41 vampire bats, sample size for diet and immune assays varied as we did not obtain sufficient hair and blood from all bats. We acquired stable isotopes for 36 bats, leucocyte profiles for 32 bats, BKA for 21 bats and IgG OD for 22 bats. Complete immune profiles (absolute leucocytes, BKA and IgG OD) were available for 17 bats.

We first used linear models to test relationships between bat diet and THg. We compared a set of models with the proportion of diet comprising domestic prey; univariate, additive and interactive effects of age, sex, site and year; and an intercept-only model (electronic supplementary material, table S1). More complex models were not considered to keep the number of models (*R* = 24) and coefficients (*k* = 1–4) small relative to our sample size [63]. We compared models with Akaike information criterion corrected for small sample size (AICc) applied to a reduced dataset (*n* = 34) owing to missing values. We next performed model averaging with the *MuMIn* package to compute standardized coefficients and 95% confidence intervals across models within six ΔAICc [64,65].

To test whether hair THg predicts bat immune profiles, we used univariate and multivariate frameworks. We first tested for associations between THg and each immune parameter (absolute leucocyte counts, NL ratios, BKA, IgG OD). Absolute leucocyte counts were analysed using generalized linear models with a Tweedie distribution and log link function to accommodate lower bounds at zero [66], especially as monocytes, eosinophils and basophils showed strong zero inflation. NL ratios were modelled with linear regression. BKA was modelled with variable dispersion beta regression to control for assay plate [60], and IgG OD was modelled using a linear mixed effects model with assay plate included as a random effect using the *nlme* package [62]. We accounted for the false discovery rate from these multiple tests by applying the Benjamini--Hochberg correction to *p-*values [67]. As components of the immune system do not act independently, we next performed a principal components (PC) analysis on absolute leucocyte counts, BKA and IgG OD (*n* = 17 with complete data), with variables centred and scaled to have unit variance. PC1 and PC2 explained 61% of the variation in immunity, and no other axis was supported by parallel analysis. We first performed a permutation multivariate analysis of variance (PERMANOVA) using Euclidean distances of immune variables in the PC analysis to test the relationship between THg and immune profiles with the *vegan* package [68]. We then used two linear regressions to test associations between THg and both PC1 and PC2.

## 3. Results

We sampled 27 bats at Ka'Kabish (*n* = 16 in 2014, *n* = 11 in 2015) and 14 bats at LAR (*n* = 7 in 2014, *n* = 7 in 2015). Our sample mostly composed of male (*n* = 34) and adult (*n* = 30) individuals. Hair THg ranged from 0.02 to 3.03 mg kg^−1^ (0.25 ± 0.59), invariably at least an order of magnitude below toxicity thresholds (10 mg kg^−1^); only three bats had THg above 1 mg kg^−1^.

### 3.1. Predictors of Hg exposure

**Figure 2.**
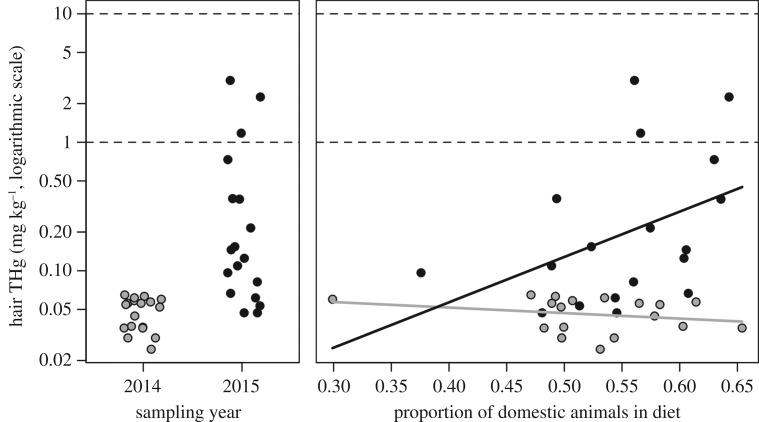
Relationships between year, bat diet and hair THg for the top-supported linear models. Solid lines show the fitted values, and dashed lines show THg thresholds of 1 mg kg^−1^ and 10 mg kg^−1^. THg values are shown on a log scale.

The best predictors of this variation in THg were bat diet, sampling year and their interaction (table 1), whereas sampling site, bat age and sex had weak effects (electronic supplementary material, figure S1, table S1). Specifically, year and the proportion of domestic animals in diet had relative importance of 1.00 and 0.45 and were positively associated with THg, although the confidence intervals for their averaged coefficients overlapped with zero (electronic supplementary material, figure S1). Only the coefficient for the interaction between bat diet and year differed significantly from zero (relative importance = 0.31; electronic supplementary material, figure S1). The model with this interaction was the most competitive (*w_i_* = 0.31) and explained 44% of variation in THg (table 1). Applying this model to a dataset with all diet and year values (*n* = 36) revealed that bats feeding more frequently on domestic animals had higher THg only in 2015 (*F*_1,32_ = 4.47, *p* = 0.04; figure 2). The increase in THg in 2015 occurred in both colonies (electronic supplementary material, figure S2).

**Table 1.** Subset of candidate linear models predicting log hair THg concentrations (mg kg^−1^). Competing models are ranked by ΔAICc with the number of estimated coefficients (*k*), Akaike weights (*w_i_*) and adjusted *R*^2^ statistic. Only models within 10 ΔAICc are shown.

log hair THg model	*k*	ΔAICc	*w_i_*	*R*^2^
∼domestic + year + domestic:year	4	0.00	0.31	0.44
∼year	2	1.09	0.18	0.36
∼year + site	3	1.42	0.15	0.38
∼domestic + year	3	1.74	0.13	0.38
∼year + site + year:site	4	2.09	0.11	0.40
∼year + age + year:age	4	2.20	0.10	0.40
∼age + site + age:site	4	8.96	<0.01	0.27
∼domestic + age	3	8.99	<0.01	0.24
∼domestic + age + site	4	9.91	<0.01	0.25

### 3.2. Immunological correlates of THg

**Figure 3.**
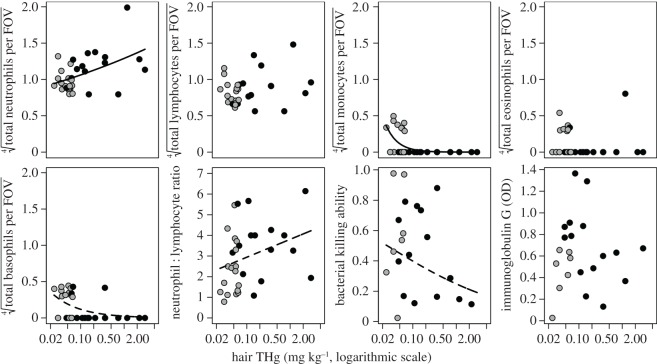
Univariate tests of the relationship between hair THg and immune parameters (absolute leucocyte counts, NL ratios, BKA, IgG OD). Solid lines indicate statistically significant fits from generalized linear models, linear regressions or variable dispersion beta regression. Dashed lines show fits from models with suggestive associations but non-significant effects after adjustment for multiple comparisons (*p* < 0.15). THg values are shown on a log scale. Bats sampled in 2014 are shown in grey and those sampled in 2015 are shown in black.

Relationships between vampire bat hair THg and immune function varied (figure 3). Bats with greater THg had more neutrophils (*β* = 0.08, *t* = 3.03, *p* = 0.04) and fewer monocytes (*β* = –1.58, *t* = –2.76, *p* = 0.04). THg had no relationship with the number of lymphocytes (*β* = 0.05, *t* = 1.36, *p* = 0.25) nor eosinophils (*β* = –0.02, *t* = –0.06, *p* = 0.95). THg was marginally negatively correlated with the number of basophils (*β* = –0.69, *t* = –2.12, *p* = 0.08) and weakly positively correlated with NL ratios (*β* = 0.38, *t* = 1.80, *p* = 0.15). THg showed a marginal negative relationship with BKA after accounting for variable dispersion by assay plate (*β* = –0.30, *z* = –2.10, *p* = 0.08). THg was not associated with IgG OD (*β* = –0.02, *t* = –0.35, *p* = 0.84).

**Figure 4.**
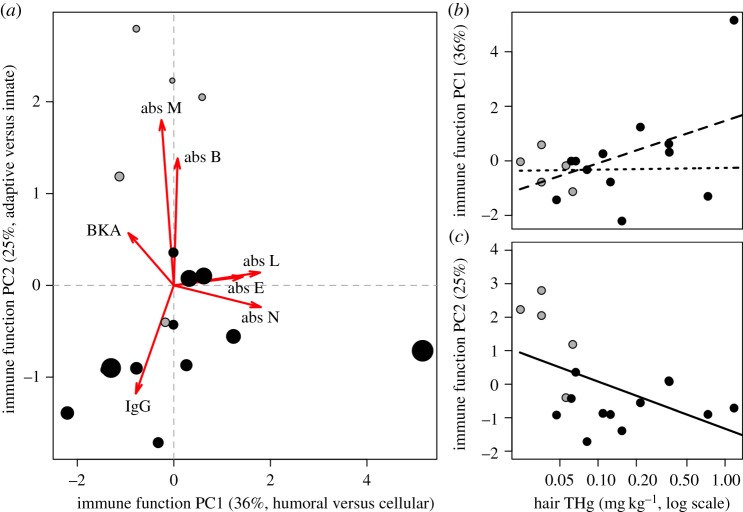
Multivariate analysis of associations between hair THg and bat immune profiles. (*a*) Biplot of the first two PCs on seven measures of immune function, with arrows indicating PC loadings and points scaled by THg. (*b*,*c*) Linear relationships between THg and the first two PCs, with the dotted line showing model fit after outlier removal. THg values are shown on a log scale. Bats sampled in 2014 are shown in grey and those sampled in 2015 are shown in black.

In our multivariate analysis, PC1 and PC2 explained 61% of the variation in immune variables (figure 4*a*). PC1 was loaded positively by absolute neutrophils (0.57), absolute lymphocytes (0.56), absolute eosinophils (0.45) and absolute basophils (0.03), and loaded negatively by absolute monocytes (–0.08), BKA (–0.30) and IgG OD (–0.26); PC1 thus represents an axis describing investment in humoral versus cellular immune function. PC2 was loaded positively by absolute lymphocytes (0.05), absolute monocytes (0.68), absolute eosinophils (0.04), absolute basophils (0.54) and BKA (0.21), and loaded negatively by absolute neutrophils (–0.09) and IgG OD (–0.43); this axis better describes variation in innate versus adaptive immune function. The PERMANOVA suggested an overall association between THg and bat immune profiles (*F*_1,15_ = 2.16, *p* = 0.06, *R*^2^ = 0.13; figure 4*a*). Independent linear regressions showed that THg correlated positively with PC1 (*F*_1,15_ = 4.25, *p* = 0.06, *R*^2^ = 0.17); however, this relationship disappeared following the removal of an outlier with high PC1 (*G* = 3.25, *U* = 0.30, *p* < 0.001; figure 4*b*; dotted line). PC2 declined with THg (*F*_1,15_ = 5.34, *p* = 0.04, *R*^2^ = 0.21; figure 4*c*).

Owing to the strong effect of sampling year on THg, we repeated the above analyses to assess inter-annual variation in immunity that could correspond to annual differences in bat THg. The relationships between year and immunity were similar to those between THg and immunity (electronic supplementary material, table S2, point colour in figures 3 and 4); vampire bats sampled in 2015 had more neutrophils, fewer monocytes, lower BKA and lower PC2.

## 4. Discussion

We here demonstrate variation in Hg exposure in vampire bats that correlates with several immune measures, even at levels typically considered to be non-toxic. Because these immune functions can be important for defending against bacterial pathogens in particular, our findings suggest chronic Hg exposure could enhance vampire bat susceptibility to such infections.

### 4.1. Vampire bat THg concentrations

Low THg concentrations in vampire bats were surprising, given that this species can consume up to its body weight in blood [28]. THg values observed in vampire bats were higher than those in frugivores (e.g. *Megaerops ecaudatus*, *Cynopterus horsfieldii*) and some insectivores (e.g. *Myotis grisescens*, *Lasiurus borealis*); however, most insectivores had THg orders of magnitude above vampire bats (e.g. *Myotis lucifugus*, *Hipposideros dyacorum*; table 2). This intermediate placement of vampire bats between frugivorous and insectivorous species highlights that these animals are only one trophic level above terrestrial herbivores, a relatively weak source of Hg. THg concentrations in blood are also frequently lower than those in tissue [69], which would likewise lead to lower dietary exposure. Comparative studies integrating THg data across bat species, particularly *Diphylla ecaudata* and *Diaemus youngi* and additional species in the Neotropics, Africa and Asia, could broadly examine diet differences as a driver of Hg exposure.

**Table 2.** Comparison of mean hair THg (μg g^−1^, mg kg^−1^, ppm) between vampire bats and select bat species from previously published studies.

species	THg	diet	study
*Myotis lucifugus*	67.55	insectivore	[16]
*Eptesicus fuscus*	19.48	insectivore	[69]
*Hipposideros dyacorum*	9.53	insectivore	[15]
*Myotis leibii*	5.30	insectivore	[13]
*Hipposideros doriae*	5.14	insectivore	[15]
*Myotis septentrionalis*	4.40	insectivore	[13]
*Hipposideros bicolor*	2.29	insectivore	[15]
*M. lucifugus*	1.50	insectivore	[13]
*E. fuscus*	1.50	insectivore	[13]
*M. septentrionalis*	0.60	insectivore	[14]
*Desmodus rotundus*	0.25	sanguivore	this study
*Myotis grisescens*	0.12	insectivore	[14]
*E. fuscus*	0.10	insectivore	[14]
*Lasiurus borealis*	0.05	insectivore	[14]
*Lasiurus cinereus*	0.02	insectivore	[14]
*Megaerops ecaudatus*	0.02	frugivore	[15]
*Cynopterus horsfieldii*	0.01	frugivore	[15]

### 4.2. Drivers of Hg exposure

Long-term feedings patterns and sampling year were the best predictors of vampire bat THg. The positive relationship between feeding on domestic prey and THg suggests several sources of Hg contamination related to agriculture. A local point source of Hg seems unlikely, as THg did not differ between sampling sites (electronic supplementary material, table S1, figure S1). Agricultural sources of Hg that affect the broader region are more probable, given that both roosts were located within a matrix of cattle pasture and agricultural fields (figure 1*a*) and most bats fed primarily on domestic animal prey. Corroborating this, some of the highest Hg concentrations in Morelet's crocodile (*Crocodylus moreletii*) eggs and tail scutes in Belize were found near cattle ranches [37,38]. Contamination of livestock with Hg could stem from atmospheric deposition [24], slash-and-burn practices [25], agrochemical use [26] or feed [27]. Intensified agricultural activity in this region of Belize [70] could also explain the positive correlation between THg and foraging on domestic animals during 2015 but not 2014; however, the underlying driver of this temporal variation in THg is unknown. A highly localized shift in land use is an unlikely explanation for higher THg in 2015 given that this increase occurred in both sites (electronic supplementary material, figure S2); thus this temporal trend might better reflect more widespread regional shifts in agricultural activity. Alternatively, drought can increase bioavailable Hg [71] and thus could explain higher THg concentrations of bats in 2015, though support for this in our system is mixed. Orange Walk District received below-average rainfall in April 2014 compared with April 2015 but received above-average rainfall in May 2015 compared with May 2014 (electronic supplementary material, figure S3). More generally, however, coherence between the temporal differences in THg and immune profiles provides evidence that the observed THg variation corresponds to underlying Hg exposure that could be potentially driven by local environmental change. Further longitudinal study could help determine if vampire bat exposure to Hg is increasing over time and driven by land conversion practices or shifts in abiotic factors.

The positive relationship between bat diet and THg also suggests feeding on livestock as a direct route of Hg exposure. While feeding on blood from terrestrial herbivores may be a low source of Hg compared with feeding on invertebrates, foraging on domestic prey reared in contaminated habitats could expose vampire bats to higher Hg. For example, livestock in areas of Brazil with soil and water contaminated from gold mining displayed elevated blood Hg concentrations [18] that exceeded toxicity thresholds for dietary intake [72,73]. To better understand transport of Hg into vampire bats, future work could assess Hg concentrations in prey and in habitat substrates. Comparative studies of locations with and without point sources of Hg contamination—such as regions of the Amazon where gold mining is absent or occurs frequently [19]—could both accomplish these goals and identify additional risk factors for Hg exposure that were limited by the small scale of our study (e.g. weak relationships with bat sex and age).

### 4.3. Immunological correlates of THg

Our data show that vampire bats with higher hair THg tended to have greater numbers of neutrophils, fewer monocytes, higher signatures of chronic stress and reduced *E. coli* killing ability. Bats with more THg did not differ in their investment in cellular versus humoral immunity but showed lower innate functions. These same trends were also reflected between bats sampled in 2014 and 2015 (when THg concentrations were greatest), providing additional support for annual variation in bat Hg exposure influencing observed immune patterns.

Neutrophils are the primary leucocytes involved in defence against bacteria and fungi, and increased numbers can indicate acute infection. In contrast with our findings, laboratory studies show that Hg reduces neutrophil counts [74,75]. This discrepancy may reflect negative feedbacks between Hg exposure, immune impairment, greater susceptibility to infection, and increased inflammatory response [76]. Specifically, impaired neutrophil production under chronic Hg exposure could increase susceptibility to bacterial infection, in turn causing inflammation and elevating neutrophil counts [77]. The negative (though weak) correlation between THg and BKA also supports this idea that Hg exposure increases bat susceptibility to bacteria. Yet while plasma defences against *E. coli* depend on complement protein [55], effects of Hg on complement remain mostly unknown [78]. An alternative explanation for the positive relationship between Hg and neutrophil counts is that long-term elevations in glucocorticoid hormones associated with chronic stress (such as prolonged heavy metal exposure) could cause neutrophilia [53,79]. Supporting this, we observed a marginal positive relationship between THg and NL ratios. Another explanation for these results could more directly involve dietary shifts from agricultural change; for example, increased feeding on livestock could alter bat immune function owing to changes in nutrition, exposure to antibiotics and new pathogens, and crowding [22,80]. While our small sample size and number of sites precluded more robust analyses of how THg correlates with bat immunity (e.g. interactions with age or confounding with diet), the consistency of how THg is associated with innate immunity argues against such relationships arising spuriously.

Relationships between Hg exposure and innate immunity were observed at very low THg concentrations, whereas negative health effects of Hg have been primarily observed at toxicity thresholds of 10 mg kg^−1^ [16,81] or subclinical thresholds of 5 mg kg^−1^ [72,82]. For example, such concentrations impaired cellular immunity in zebra finches (*Taeniopygia guttata* [83]) and black-footed albatross (*Phoebastria nigripes* [84]). While immune impairments have occurred at lower concentrations in wildlife, such as at 2 mg kg^−1^ in grebes (*Aechmophorus* spp. [77]) and even at 0.6 mg kg^−1^ in American kestrels (*Falco sparverius* [85]), the sublethal thresholds observed here (e.g. 0.1–0.2 mg kg^−1^ in the multivariate analysis) raise the hypothesis that bat innate immunity may be especially susceptible to environmental assaults and, in particular, heavy metal contamination.

By contrast, the lack of an correlation between THg and both the lymphocyte count and IgG levels contrasts with a large body of research in mice and humans showing that Hg exposure can induce autoimmune syndromes consisting of B- and T-lymphocyte activation and increased immunoglobulin concentrations [86,87]. However, Hg has also been found to delay the onset of B-lymphocyte proliferation in response to antigen and to inhibit antibody synthesis [83,88]. That we did not detect associations between THg and these measures in either direction could imply that Hg concentrations were too low to influence adaptive immune function [89].

## 5. Conclusion

To our knowledge, this is the first study to assess Hg concentrations in a Neotropical bat species or to assess correlations between Hg and immunity in bats. Our data suggest hair concentrations of THg far below known thresholds for toxicity and adverse health effects may negatively affect the immunology of vampire bats in ways that could increase susceptibility to bacterial infections. While *D. rotundus* is the primary reservoir of rabies virus in Latin America [29], this species can also be infected by bacteria such as *Bartonella* spp. and *Leptospira* spp. [90,91]. As improved feeding success in agricultural habitat could also promote transmission by increasing bat contact and population density [22,92], greater susceptibility from chronic Hg exposure could enhance spread of bacterial infections and pose spillover risks to humans and livestock [80,93].

Owing to these epidemiological risks, future work should focus on geographical areas of high Hg contamination (e.g. regions where gold mining is practised) to evaluate effects of a wider range of Hg exposure levels on bat immunity and pathogen diversity. Such studies could also evaluate sympatric bat species with different feeding ecologies to assess routes of Hg exposure and whether bat immunity is generally as unusually sensitive to Hg exposure as we have here documented for vampire bats. Quantifying how Hg exposure affects immunity and infection in temperate bat species is another important aim to inform conservation efforts against white-nose syndrome. Lastly, our correlational findings will require captive experimental trials to establish causality between Hg exposure and altered bat immunity and to establish thresholds for such changes. These potential consequences for human health and bat conservation underscore the need to better understand effects of Hg on bat immunity and infectious disease.

## Supplementary Material

Predictors and immunological correlates of sublethal mercury exposure in vampire bats: Online Supplemental Information

## Data accessibility

Data are available in the Dryad Digital Repository: http://dx.doi.org/10.5061/dryad.65p70 [94].
