# Supplementary material for: Predictors and immunological correlates of sublethal mercury exposure in vampire bats
Source: R Soc Open Sci. 2017 Apr 19;4(4):170073. doi: 10.1098/rsos.170073 (PMC5414270; doi:10.1098/rsos.170073)
Supplement: Predictors and immunological correlates of sublethal mercury exposure in vampire bats: Online Supplemental Information [file rsos170073supp1.pdf]

**Predictors and immunological correlates of sublethal mercury exposure in vampire bats: Online Supplemental Information**

Daniel J. Becker, Matthew M. Chumchal, Alexandra B. Bentz, Steven G. Platt, Gábor Á. Czirják, Thomas R. Rainwater, Sonia Altizer, and Daniel G. Streicker

**S1. Model comparison and averaging**
**S2. Site and year effects on THg**
**S3. Immunity and year relationships**
**S4. Temporal patterns in rainfall**

**S1. Model comparison and averaging**

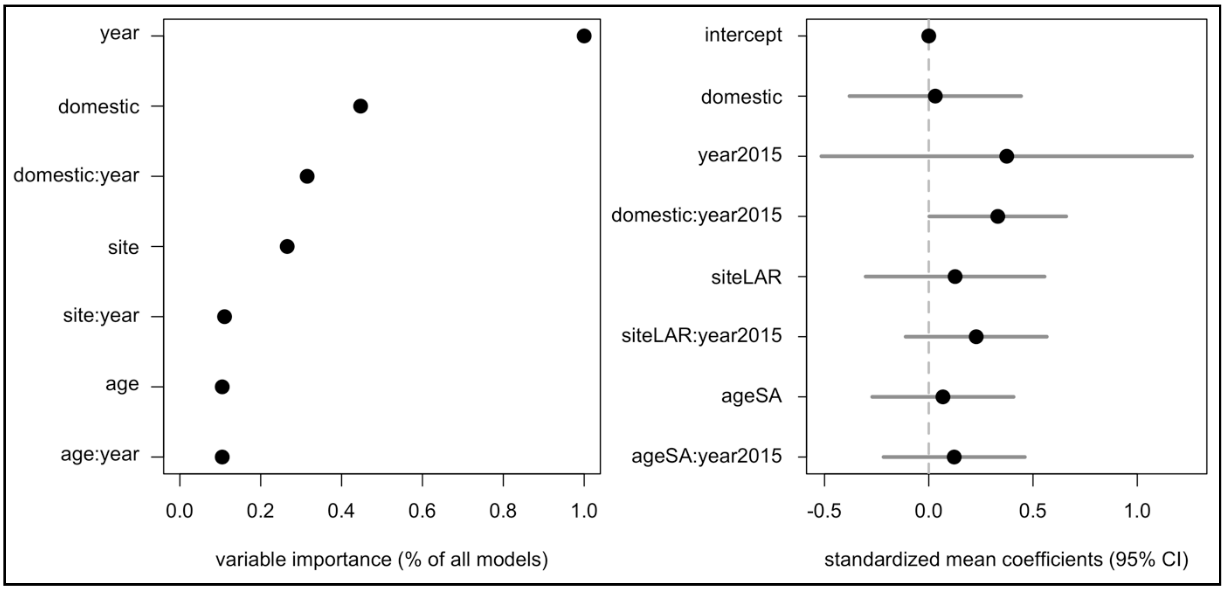


Figure S1. Relative importance of variables considered in model comparison, with log THg (mg/kg) as the dependent variable in linear regressions (A). Averaged coefficients and 95% confidence intervals for all terms considered, standardized by the ratio of standard deviations of log THg and each dependent variable (B). The dashed line represents *β*=0 (no effect on THg).

Table S1. All candidate linear models predicting log hair THg concentrations (mg/kg). Competing models are ranked by ΔAICc along with the number of estimated coefficients (*k*), the Akaike weights ($w_i$), and the adjusted $R^2$ statistic.

| **Log hair THg model** | ***k*** | **ΔAICc** | $w_i$ | $R^2$ |
|---|---|---|---|---|
| ~ domestic + year + domestic:year | 4 | 0.00 | 0.31 | 0.44 |
| ~ year | 2 | 1.09 | 0.18 | 0.36 |
| ~ year + site | 3 | 1.42 | 0.15 | 0.38 |
| ~ domestic + year | 3 | 1.74 | 0.13 | 0.38 |
| ~ year + site + year:site | 4 | 2.09 | 0.11 | 0.40 |
| ~ year + age + year:age | 4 | 2.20 | 0.10 | 0.40 |
| ~ age + site + age:site | 4 | 8.96 | <0.01 | 0.27 |
| ~ domestic + age | 3 | 8.99 | <0.01 | 0.24 |
| ~ domestic + age + site | 4 | 9.91 | <0.01 | 0.25 |
| ~ domestic + age + domestic:age | 4 | 10.54 | <0.01 | 0.24 |
| ~ domestic + age + sex | 4 | 11.45 | <0.01 | 0.22 |
| ~ age | 2 | 12.00 | <0.01 | 0.13 |
| ~ age + site | 3 | 12.06 | <0.01 | 0.17 |
| ~ domestic + site | 3 | 13.63 | 0.00 | 0.13 |
| ~ site | 2 | 13.79 | 0.00 | 0.09 |
| ~ age + sex + age:sex | 4 | 14.25 | 0.00 | 0.15 |
| ~ age + sex | 3 | 14.53 | 0.00 | 0.10 |
| ~ domestic + site + domestic:site | 4 | 14.72 | 0.00 | 0.14 |
| ~ domestic | 2 | 14.85 | 0.00 | 0.06 |
| ~ 1 | 1 | 15.56 | 0.00 | 0/00 |
| ~ domestic + site + sex | 4 | 16.35 | 0.00 | 0.10 |
| ~ domestic + sex + domestic:sex | 4 | 16.69 | 0.00 | 0.09 |
| ~ domestic + sex | 3 | 17.29 | 0.00 | 0.03 |
| ~ sex | 2 | 17.95 | 0.00 | 0.00 |

**S2. Site and year effects on THg**

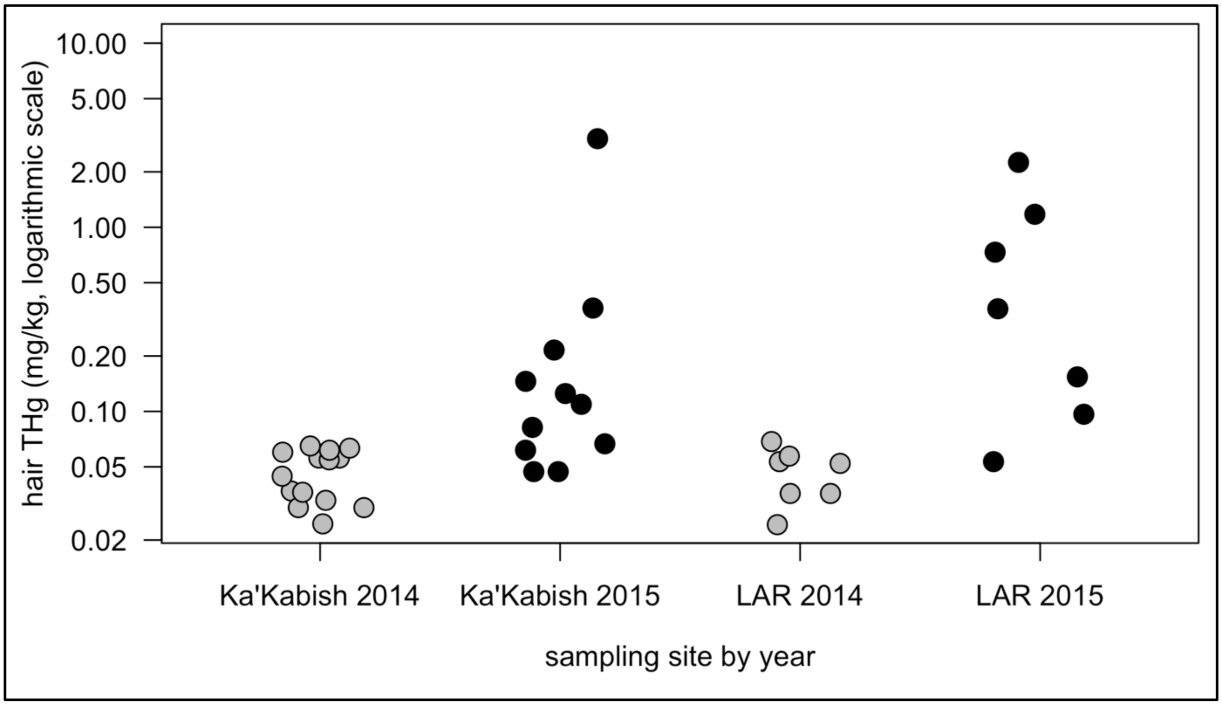


Figure S2. Relationship between sampling site, year, and log bat THg. Points are jittered to avoid overlap; bats sampled in 2014 are shown in grey and those sampled in 2015 are shown in black. A linear regression showed no significant interaction between site and year ($F_{1,31}$=1.88, $p$=0.18).

## S3. Immunity and year relationships

Table S2. Relationships between sampling year and vampire bat immune function.

| **Immune models** | **Test statistic** | ***p* value** |
|---|---|---|
| Absolute neutrophils ~ year | 0.2137* | 0.0048 |
| Absolute lymphocytes ~ year | 0.1053* | 0.2355 |
| Absolute monocytes ~ year | -21.5625* | 0.9923 |
| Absolute eosinophils ~ year | -2.2012* | <0.001 |
| Absolute basophils ~ year | -0.9966* | 0.0677 |
| NL ratio ~ year | 1.2013* | 0.0219 |
| Bacterial killing ability ~ year | -1.2504* | <0.001 |
| Immunoglobulin G OD ~ year | 0.2283* | 0.1242 |
| PERMANOVA ~ year | 0.1433** | 0.0302 |
| Immune PC1 ~ year | 0.4372* | 0.6209 |
| Immune PC2 ~ year | -2.2012* | <0.001 |

* Test statistic is $\beta$ coefficient from linear model, GLM, beta regression, or LMM
** Test statistic is $R^2$ from PERMANOVA

## S4. Temporal patterns in rainfall

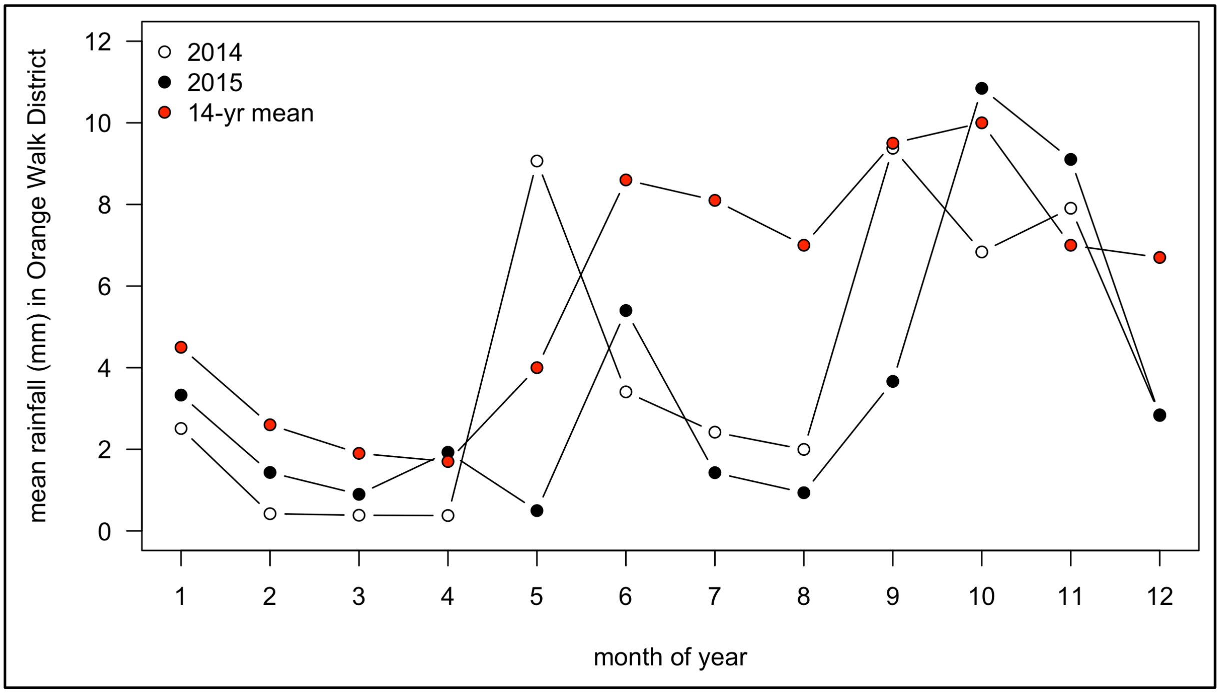


Figure S3. Temporal patterns of rainfall in Orange Walk District, Belize during 2014 and 2015. Data are available from the National Meteorological Service of Belize.
